# XPO1 inhibitor KPT-330 synergizes with Bcl-xL inhibitor to induce cancer cell apoptosis by perturbing rRNA processing and Mcl-1 protein synthesis

**DOI:** 10.1038/s41419-019-1627-9

**Published:** 2019-05-21

**Authors:** Zhi-Chuan Zhu, Ji-Wei Liu, Can Yang, Miao Zhao, Zhi-Qi Xiong

**Affiliations:** 1 Institute of Neuroscience, State Key Laboratory of Neuroscience, CAS Center for Excellence in Brain Science and Intelligence Technology, Chinese Academy of Sciences, Shanghai, China; 2 Shanghai Mental Health Center, Shanghai Jiao Tong University School of Medicine, Shanghai, China; 3 University of Chinese Academy of Sciences, Beijing, China; 4 Department of Neurology and Institute of Neurology, The First Affiliated Hospital of Fujian Medical University, Fuzhou, China; 5 School of Life Science and Technology, ShanghaiTech University, Shanghai, China

**Keywords:** RNA, Targeted therapies, Apoptosis, Transport receptors

## Abstract

XPO1 (exportin1) mediates nuclear export of proteins and RNAs and is frequently overexpressed in cancers. In this study, we show that the orally bioavailable XPO1 inhibitor KPT-330 reduced Mcl-1 protein level, by which it synergized with Bcl-xL inhibitor A-1331852 to induce apoptosis in cancer cells. KPT-330/A-1331852 combination disrupted bindings of Mcl-1 and Bcl-xL to Bax, Bak, and/or Bim, elicited mitochondrial outer membrane permeabilization, and triggered apoptosis. KPT-330 generally mitigated mRNA expression and protein synthesis rather than mRNA nuclear export or protein stability of Mcl-1. KPT-330 inhibited mTORC1/4E-BP1 and Mnk1/eIF4E axes, which disrupted the eIF4F translation initiation complex but was dispensable for Mcl-1 reduction and KPT-330/A-1331852 combination-induced apoptosis. Mature rRNAs are integral components of the ribosome that determines protein synthesis ability. KPT-330 impeded nucleolar rRNA processing and reduced total levels of multiple mature rRNAs. Reconstitution of XPO1 by expressing degradation-resistant C528S mutant retained rRNA amount, Mcl-1 expression, and Bcl-xL inhibitor resistance upon KPT-330 treatment. KPT-330/A-1331852 combination suppressed growth and enhanced apoptosis of non-small cell lung cancer xenografts. Therefore, we clarify the reason of apoptosis resistance of cancer cells to XPO1 inhibition and develop a potential strategy for treating solid tumors.

## Introduction

Exportin1 (XPO1, also known as chromosomal maintenance region 1, or CRM1) mediates nuclear export of proteins and RNAs, and ribosome biogenesis, which are important for cancer growth and survival^1^. *XPO1* is frequently amplified or mutated in several hematological and solid tumors. XPO1 overexpression correlates with poor prognosis in various cancers, whereas either targeting XPO1 alone by the selective inhibitors of nuclear export (SINE) or in combination with other targeted therapies or chemotherapies shows broad anticancer effect and acceptable tolerance^2–4^. SINE compounds degrade XPO1 protein by specific binding to its C528 residue in the cargo-binding groove. One of the first-generation orally bioavailable SINEs, KPT-330 (selinexor) is under testing in patients in 64 phase I/II/III trials (ClinicalTrials.gov), whilst the brain-associated adverse effects like anorexia and weight loss, and hematologic adverse effects like thrombocytopenia limit its dose^5^. The second-generation SINE, KPT-8602 has proven its activity against hematological malignancies, with improved tolerability than KPT-330 owing to its lower brain penetration in preclinical animal models^6,7^.

The balance between the antiapoptotic (Bcl-2, Bcl-xL, Mcl-1, and less studied Bcl-W and BFL-1) and proapoptotic Bcl-2 family proteins (Bax, Bak, and BH3 domain-only proteins) determines the activity of mitochondrial apoptotic signaling^8^. The functional redundancy of antiapoptotic proteins safeguards cancer cells from apoptotic induction when some of the proteins are compromised. Whereas high Bcl-2 expression dominates the survival of some liquid tumors making targeting Bcl-2 sufficient to kill them^9,10^, Bcl-xL and Mcl-1 often act as double insurance for solid tumor survival increasing the apoptotic threshold and entailing dual targeting for apoptosis induction^10–13^. The development of the dual Bcl-2/Bcl-xL inhibitor ABT-263 ended up in vain due to thrombopenia resulted from Bcl-xL inhibition. However, the Bcl-xL-selective inhibitors A-1155463 and A-1331862 demonstrated tolerability and efficacy in preclinical solid tumor models^14^. Mcl-1 is a short-lived protein that is vulnerable to suppression of protein expression on the transcriptional, post-transcriptional, translational, or post-translational levels^11,15–17^. Recently, Mcl-1-selective inhibitors evolved and one of them showed exceptional anticancer efficacy^12,18^. Furthermore, it was demonstrated that SINE compounds including KPT-185, KPT-276, and KPT-330 downregulated Mcl-1 protein^19–21^, but the underlying mechanism and function of Mcl-1 upon SINE treatment are unclear. It was hypothesized in one prior study that nuclear retention of Mcl-1 mRNA caused Mcl-1 downregulation^20^.

In this study, we investigated the effect and regulatory mechanism of KPT-330 on Mcl-1 expression and developed combination therapy to enhance the anticancer activity of KPT-330. We demonstrated that KPT-330 decreased Mcl-1 protein synthesis through mitigating rRNA processing and global protein synthesis, making cancer cells more susceptible to Bcl-xL inhibitors like A-1331852. KPT-330 synergized with A-1331852 to induced apoptosis in a range of cancer cells in vitro and suppressed tumor growth in a non-small cell lung cancer (NSCLC) model.

## Results

### XPO1 and Bcl-xL inhibitors synergistically induce apoptosis in cancer cells

**Fig. 1:**
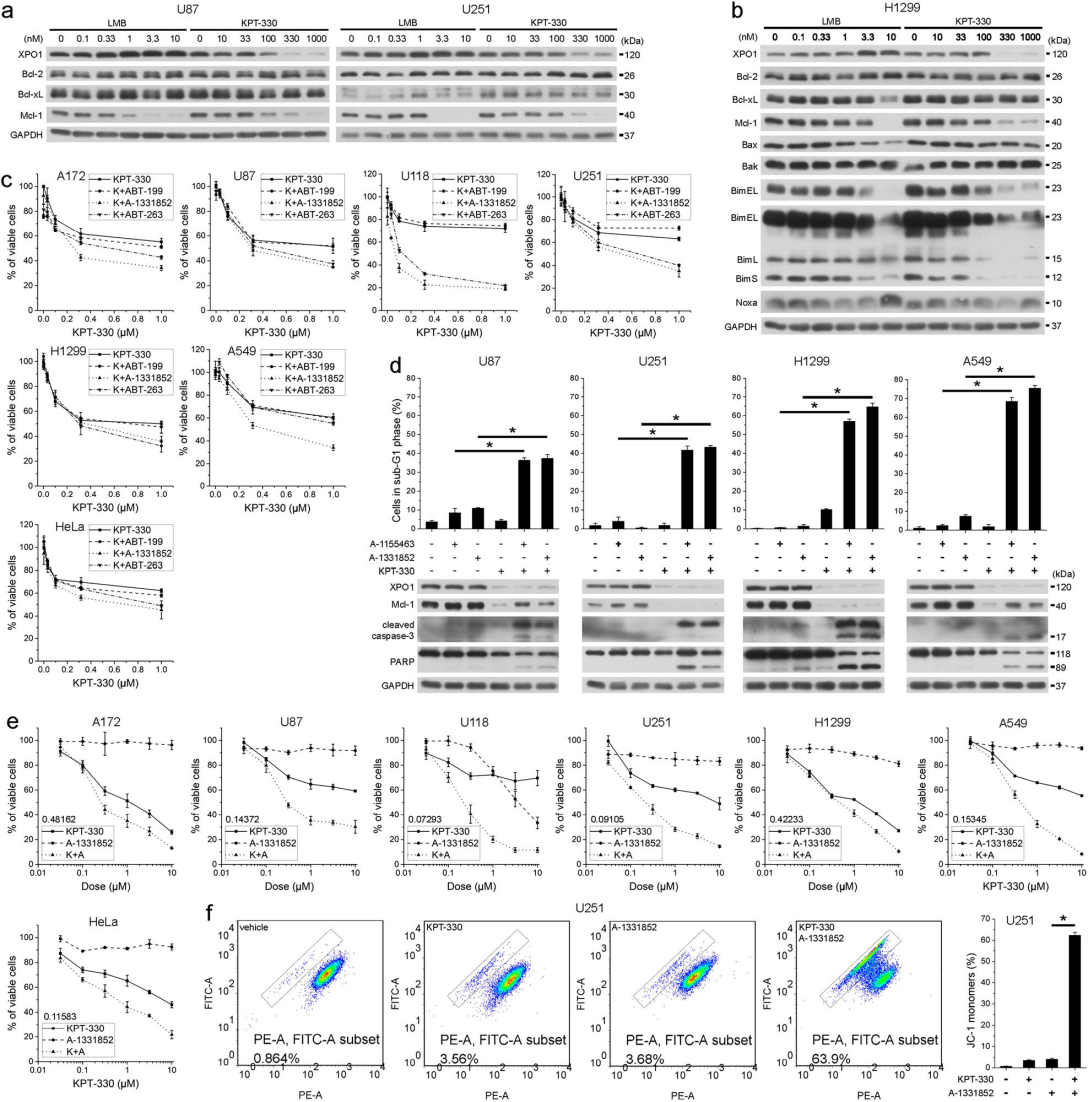
XPO1 and Bcl-xL inhibitors synergistically induce apoptosis in cancer cells. **a**, **b** U87, U251, and H1299 cells were treated with indicated dose of LMB or KPT-330 for 48 h and subjected to western blot. **c** Four glioblastoma, two NSCLC, and one cervical cancer cell lines were treated with indicated dose of KPT-330 together with 1 μM of ABT-199, A-1331852, or ABT-263 for 48 h. Cell viability was measured by the MTT assay (mean ± SD, *n* = 3). **d** U87, U251, H1299, and A549 cells were treated with KPT-330 (1 μM) for 24 h and further with A-1155463 (1 μM) or A-1331852 (1 μM) for 24 h, then subjected to western blot or flow cytometry analysis for the sub-G1 phase (mean ± SD, *n* = 3). *P < 0.05. **e** Cell lines in **c** were treated with indicated dose of KPT-330 or A-1331852 or both for 48 h. Cell viability was measured by the MTT assay. Combination index (CI) at ED50 was calculated by ComboSyn software with the Chou-Talaley equation (mean ± SD, *n* = 3). **f** The mitochondrial membrane potential in U251 cells treated as in **d** was measured by flow cytometry (mean ± SD, *n* = 3). *P < 0.05. GAPDH was used as the loading control

We interrogated the effect of XPO1 inhibitors on antiapoptotic Bcl-2 proteins to gain insights on the molecular mechanism conferring their inefficient apoptosis-inducing capacities. The XPO1 inhibitor leptomycin B (LMB) and KPT-330 consistently downregulated Mcl-1 but not Bcl-2 or Bcl-xL in a dose-dependent manner in U87 and U251 glioblastoma cells and H1299 NSCLC cells (Fig. 1a, b). LMB and KPT-330 also consistently downregulated Bim but not other proapoptotic Bcl-2 proteins in H1299 cells (Fig. 1b). Mcl-1 reduction correlated well with XPO1 reduction upon KPT-330 treatment (Fig. 1a, b). Although Bcl-2, Bcl-xL, and Mcl-1 have different preference in binding antiapoptotic and BH3 domain-only Bcl-2 proteins, they play redundant roles in blocking mitochondrial outer membrane permeabilization (MOMP). Therefore, Mcl-1 downregulation by XPO1 inhibitor was insufficient to induce apoptosis in cancer cells but likely made cancer cells more susceptible to inhibitors targeting of Bcl-2 and/or Bcl-xL. Indeed, in glioblastoma (A172, U87, U118, and U251), NSCLC (H1299 and A549), and cervical cancer cells (HeLa), inhibitor of Bcl-xL (A-1331852) or Bcl-2/Bcl-xL (ABT-263) but not Bcl-2 (ABT-199) further reduced the viability of cells treated with KPT-330 at the dose capable of downregulating Mcl-1 (Fig. 1c), indicating that the remaining Bcl-xL rather than Bcl-2 confers to KPT-330 resistance in these cells. Combination of KPT-330 and different Bcl-xL-selective inhibitors triggered intense apoptosis in U87, U251, H1299, and A549 cells (Fig. 1d). In glioblastoma, NSCLC, and cervical cancer cells, KPT-330 plus A-1331852 had a strong synergistic effect on viability inhibition, as evaluated by their combination index (Figs. 1e and S1). JC-1 staining showed that such combination elicited MOMP in U251 cells (Fig. 1f). These results indicate a strong synergism of XPO1 and Bcl-xL inhibitor combination in apoptosis induction in cancer cells.

### KPT-330-downregulated Mcl-1 facilitates mitochondria-mediated apoptosis upon KPT-330 and A-1331852

**Fig. 2:**
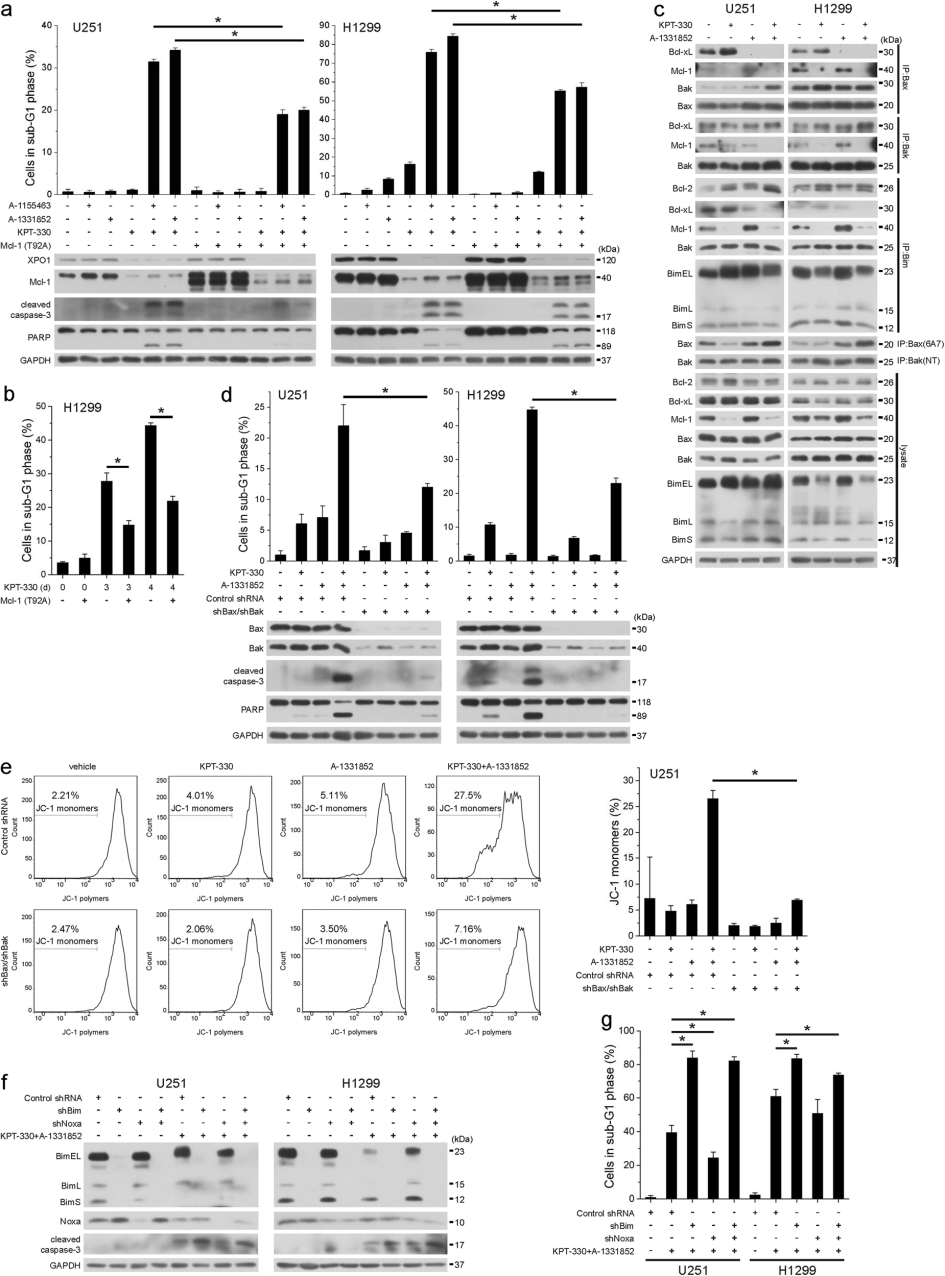
KPT-330-downregulated Mcl-1 facilitates mitochondria-mediated apoptosis upon KPT-330 and A-1331852. **a** U251 and H1299 cells expressing Mcl-1 (T92A) were treated with KPT-330 (1 μM) for 24 h and further with A-1155463 (1 μM) or A-1331852 (1 μM) for 24 h, then subjected to western blot or flow cytometry analysis for the sub-G1 phase (mean ± SD, *n* = 3). *P < 0.05. **b** H1299 cells expressing Mcl-1 (T92A) were treated KPT-330 (1 μM) for 3 or 4 days and subjected to flow cytometry analysis for the sub-G1 phase (mean ± SD, *n* = 3). *P < 0.05. **c** U251 and H1299 cells were treated with KPT-330 (1 μM) for 24 h and further with A-1331852 (1 μM) for 6 h. Total and active forms of Bax and Bak in cell lysates were immunoprecipitated followed by western blot. **d**, **e** U251 and/or H1299 cells expressing shBax and shBak were treated as in **a** and subjected to western blot (**d**), flow cytometry analysis for the sub-G1 phase (**d**), or mitochondria membrane potential (**e**) (mean ± SD, *n* = 3). *P < 0.05. **f**, **g** U251 and H1299 cells expressing shBim and/or shNoxa were treated with KPT-330 (1 μM) for 24 h and further with A-1331852 (1 μM) for 6 h or 24 h, then subjected to western blot (6 h) or flow cytometry analysis (24 h) for the sub-G1 phase (mean ± SD, *n* = 3). *P < 0.05. GAPDH was used as the loading control

Although KPT-330 treatment counteracted the effect of Mcl-1 overexpression, a slight increase of Mcl-1 level partially reversed KPT-330/A-1331852-triggered apoptosis in U251 and H1299 cells (Fig. 2a). Overexpression of Mcl-1 also made H1299 cells more resistant to long-term KPT-330 treatment (Fig. 2b). KPT-330 diminished Mcl-1 binding to Bax, Bak, and Bim while enhanced Bcl-xL binding to Bax and Bcl-2 binding to Bim possibly compensating for Mcl-1 loss. A-1331852 prevented Bax and Bim from Bcl-xL binding. Their combination thereby freed and activated both Bax and Bak (Fig. 2c). We failed to detect Bax in Bim immunoprecipitant but showed no alteration of Bak/Bim binding upon drug combination possibly due to enhanced Bim sequestration by Bcl-2 (Fig. 2c). Simultaneous knockdown of Bax and Bak reversed KPT-330/A-1331852-triggered apoptosis and MOMP in U251 cells (Fig. 2d, e). Noxa knockdown slightly reduced KPT-330/A-1331852-triggered apoptosis while Bim knockdown upregulated Noxa (at least in U251 cells) and increased such apoptosis (Fig. 2f, g), indicating the functioning of residual Mcl-1 and irrelevance of Bim in promoting apoptosis. These results suggest that Mcl-1 downregulation by KPT-330 dictates the synergism of KPT-330 and A-1331852 combination.

### KPT-330 reduces Mcl-1 mRNA expression and protein synthesis

**Fig. 3:**
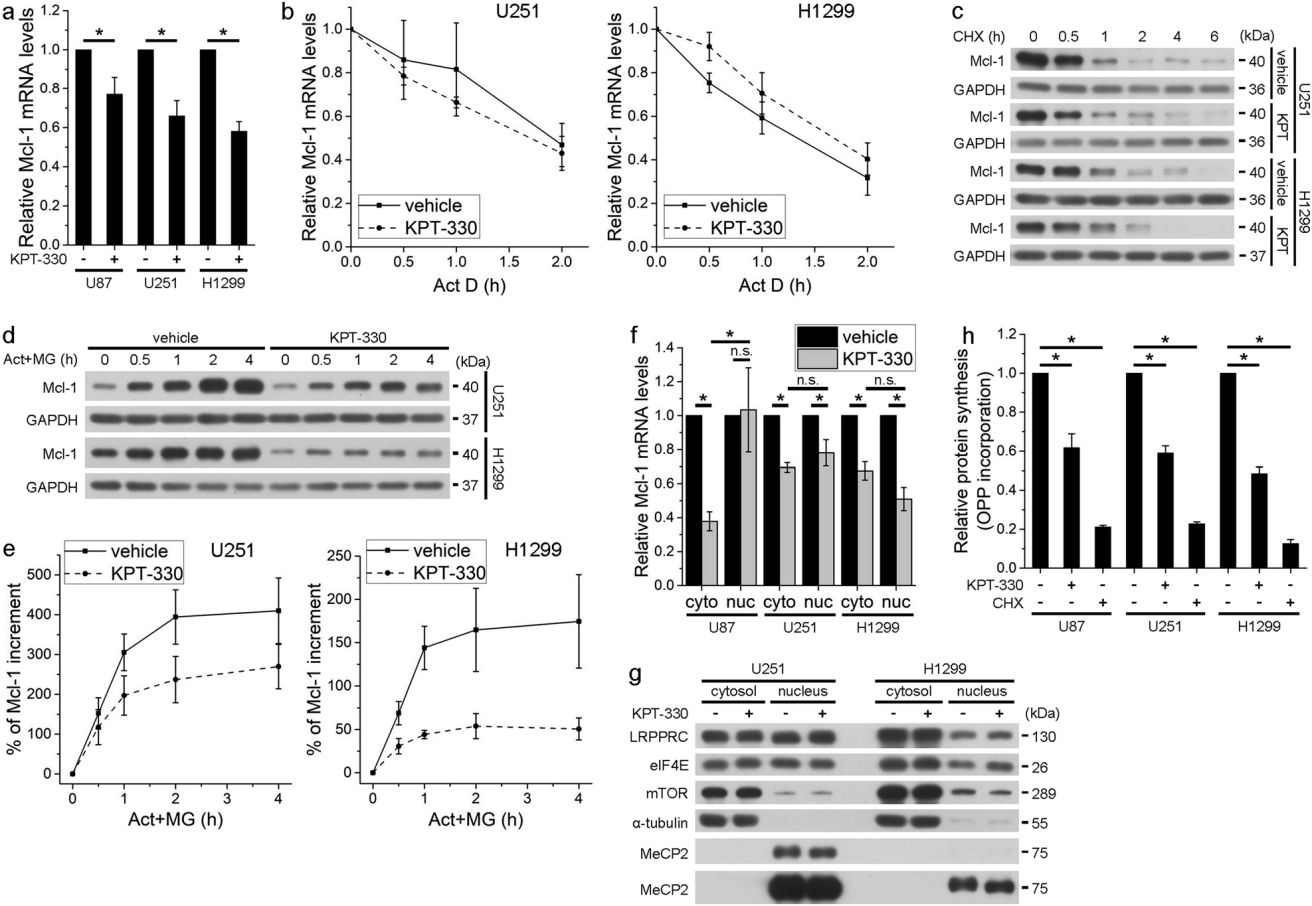
KPT-330 reduces Mcl-1 mRNA expression and protein synthesis. **a** Real-time PCR analysis of total Mcl-1 mRNA in U87, U251, and H1299 cells treated with KPT-330 (1 μM) for 24 h (mean ± SEM, *n* = 6 for U87 and U251 and *n* = 8 for H1299). *P < 0.05. **b** Real-time PCR analysis of Mcl-1 mRNA decay in U251 and H1299 cells treated with KPT-330 (1 μM) for 24 h and further with Act D (5 μg/ml) for indicated time periods (mean ± SEM, *n* = 3). *P < 0.05. **c** The CHX (100 μg/ml) pulse-chase assay in U251 and H1299 cells treated as in **a**. **d**, **e** Western blot analysis of Mcl-1 protein synthesis in U251 and H1299 cells treated with KPT-330 (1 μM) for 24 h, and further with Act D (Act) (5 μg/ml) and MG-132 (MG) (25 μM) for indicated time periods. Quantification of grayscale ratio of Mcl-1/GAPDH by Photoshop software was shown in **e** (mean ± SEM, *n* = 3). **f** Real-time PCR analysis of cytosolic and nuclear Mcl-1 mRNA in U87, U251, and H1299 cells treated as in **a** (mean ± SEM, *n* = 3). *P < 0.05. **g** Western blot analysis of indicated proteins in the cytosol and nucleus of U251 and H1299 cells treated as in **a**. **h** Nascent protein synthesis in U87, U251, and H1299 cells treated as in **a** was measured by OPP incorporation (mean ± SEM, *n* = 3). *P < 0.05. GAPDH, α-tubulin, and MeCP2 were used as the loading control of total, cytosolic, and nuclear protein, respectively

Next, we investigated how KPT-330 reduced Mcl-1 protein level. KPT-330 downregulated Mcl-1 mRNA without apparently affecting its degradation (Fig. 3a, b). KPT-330 did not influence protein degradation rate of Mcl-1 (Fig. 3c) but slowed down protein synthesis rate of Mcl-1 after blocking transcription and protein degradation by Act D and MG-132 respectively (Fig. 3d, e), reflecting a defect of Mcl-1 translation. XPO1 can export RNA into the cytosol and mRNA nuclear retention impairs its translation. However, KPT-330 did not uniformly decreased cytosolic Mcl-1 mRNA levels in all tested cells (Fig. 3f). Nor did KPT-330 affect the cytosol/nucleus distribution of LRPPRC, an XPO1 export cargo assisting eIF4E-dependent mRNA export^22^, in U251 and H1299 cells (Fig. 3g). Unexpectedly, the OPP incorporation assay demonstrated a reduced nascent synthesized protein content upon KPT-330 treatment (Fig. 3h). We further analyzed synthesis rates of some other proteins and found that the production of fast degrading proteins FLIPs and c-Myc was slowed down in U251 and H1299 cells following KPT-330 treatment (Supplementary Fig. S2a–d). These results suggest that KPT-330 impairs both mRNA expression and protein synthesis of Mcl-1.

### KPT-330-inactivated cap-dependent translation initiation is dispensable for Mcl-1 downregulation

**Fig. 4:**
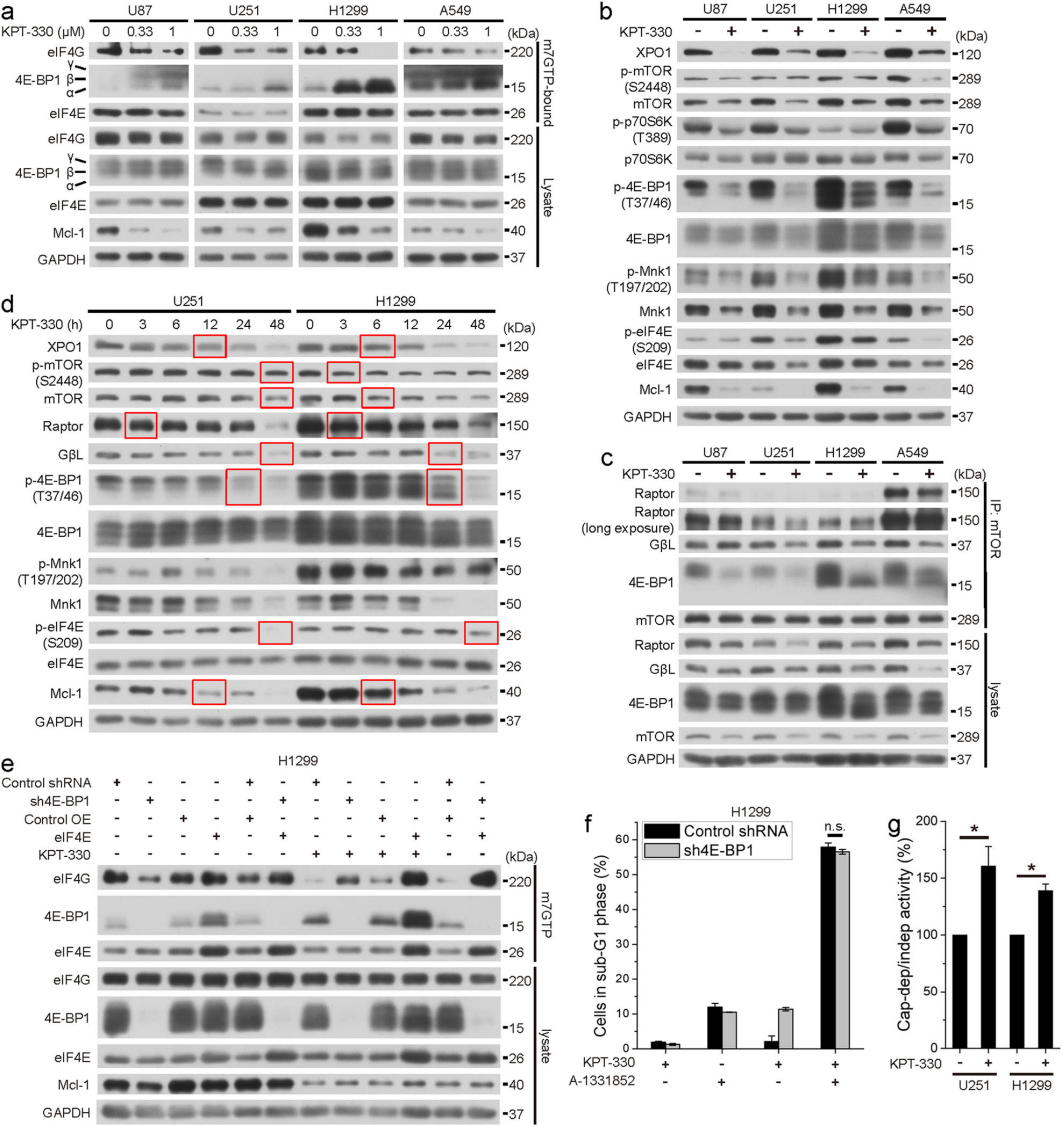
KPT-330-inactivated cap-dependent translation initiation is dispensable for Mcl-1 downregulation. **a** The cap-binding assay in U87, U251, H1299, and A549 cells treated with indicated dose of KPT-330 for 48 h. **b**, **c** Western blot of indicated proteins in U87, U251, H1299, and A549 cells treated with KPT-330 (1 μM) for 48 h. **c** Proteins in mTOR complex in cell lysates as in **b** were immunoprecipitated followed by western blot. **d** Western blot of indicated proteins in U251 and H1299 cells treated with KPT-330 (1 μM) for indicated time periods. **e** The cap-binding assay in sh4E-BP1 and/or eIF4E H1299 cells treated as in **a**. **f** H1299 cells expressing sh4E-BP1 were treated with KPT-330 (1 μM) for 24 h and further with A-1331852 (1 μM) for 24 h, then subjected to flow cytometry analysis for the sub-G1 phase (mean ± SD, *n* = 3). *P < 0.05. **g** The bicistronic luciferase reporter assay in U251 and H1299 cells treated with KPT-330 (1 μM) for 24 h (mean ± SEM, *n* = 3). *P < 0.05. GAPDH was used as the loading control

The eIF4F complex integrity is the major determinant of cap-dependent translation initiation that regulates protein synthesis. Phosphorylation of 4E-BP1 upon attenuated Akt/mTOR signaling enhances its binding to 5′ mRNA cap-bound eIF4E, which inhibits the assembly of eIF4F complex containing eIF4E and eIF4G, and ribosome recruitment to mRNA templates^13^. Moreover, phosphorylation of eIF4E at Ser209 by MAPK/Mnk1 signaling positively regulates translation initiation^23^. As the cap-binding assay showed, KPT-330 enhanced binding of 4E-BP1 and attenuated binding of eIF4G to cap-mimicking m^7^GTP-bound eIF4E in U87, U251, H1299, and A549 cells (Fig. 4a). Consistently, KPT-330 inactivated mTOR and Mnk1 signaling as revealed by downregulation of total mTOR and Mnk1 and phosphorylation of mTOR, p70S6K, 4E-BP1, Mnk1, and eIF4E (Fig. 4b). KPT-330 also downregulated mTORC1 complex member Raptor and GβL and reduced bindings of Raptor, GβL, and 4E-BP1 to mTOR in certain cell lines (Fig. 4c). However, following KPT-330 treatment, downregulation of XPO1 and concomitant Mcl-1 occurred prior to downregulation of p-4E-BP1, p-eIF4E, and GβL in U251 and H1299 cells and downregulation of (p-)mTOR in U251 cells (Fig. 4d). In H1299 cells, 4E-BP1 knockdown, and eIF4E overexpression retained eIF4E/eIF4G interaction but hardly reversed Mcl-1 expression after KPT-330 treatment (Fig.4e). Accordingly, 4E-BP1 knockdown failed to rescue KPT-330/A-1331852 combination-triggered apoptosis (Fig. 4f). Interestingly, KPT-330 augmented the ratio of cap-dependent to IRES (internal ribosome entry site)-dependent (cap-independent) translation activity, as measured by a bicistronic luciferase reporter assay (Fig. 4g), suggesting that KPT-330 suppressed both cap-dependent and independent translation initiation conferring to crippled protein synthesis ability. These results suggest that KPT-330-inhibited mTOR signaling and cap-dependent translation initiation machinery are irresponsible for Mcl-1 downregulation.

### KPT-330 inhibits rRNA processing

**Fig. 5:**
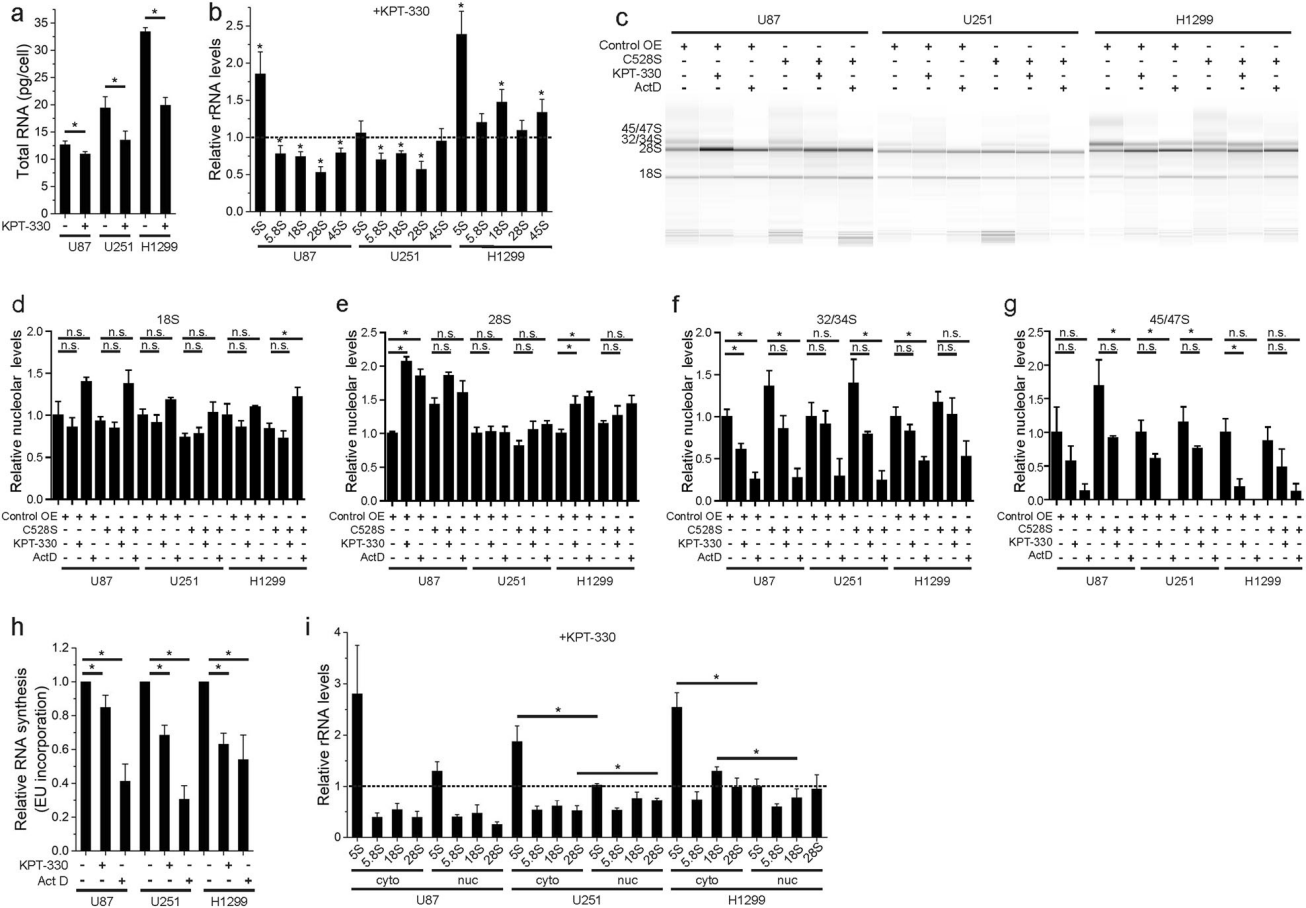
KPT-330 inhibits rRNA processing. **a** Quantification of RNA content per cell in U87, U251, and H1299 cells treated with KPT-330 (1 μM) for 24 h. (mean ± SEM, *n* = 6). *P < 0.05. **b** Real-time PCR analysis of indicated rRNAs in U87, U251, and H1299 cells treated as in **a** (mean ± SEM, *n* = 6 for U87 and U251 and *n* = 8 for H1299). *P < 0.05. **c** Nucleolar RNAs from XPO1 (C528S) expressing U87, U251, and H1299 cells treated with KPT-330 (1 μM) for 24 h or Act D (500 ng/μl) for 3 h were subjected to fragment analysis. Quantification of the relative percentage of 18S, 28S, 32/34S, and 45/47S was shown in **d**–**g** (mean ± SEM, *n* = 3–6), respectively. *P < 0.05. **h** Nascent RNA synthesis in U87, U251, and H1299 cells treated as in **a** was measured by EU incorporation (mean ± SEM, *n* = 5). *P < 0.05. **i** Real-time PCR analysis of cytosolic and nuclear rRNAs indicated in U87, U251, and H1299 cells treated as in **a** (mean ± SEM, *n* = 4 for U87 and *n* = 5 for U251 and H1299). *P < 0.05

RNA content was reduced in KPT-330-treated cells (Fig. 5a). As rRNA constitutes the majority of total cellular RNA, we evaluated levels of different rRNA in these cells. KPT-330 reduced 5.8S, 18S, and 28S rRNA, processing products of 45S pre-rRNA synthesized in the nucleolus but not 5S rRNA synthesized in the cytosol in U87 and U251 cells (Fig. 5b). In contrast, none of these rRNAs is downregulated in KPT-330-treated H1299 cells (Fig. 5b). To further clarify whether KPT-330 disrupts nucleolar rRNA synthesis or processing, the nucleoli of U87, U251, and H1299 cells treated with KPT-330 or Act D were purified and the nucleolar RNAs were extracted. Fragment analysis showed that Act D blocked the synthesis of 45/47S and 32/34S pre-rRNAs while it increased 28S rRNA. Similarly, KPT-330 treatment resulted in reduced 45/47S and 32/34S rRNAs, while it enhanced 28S in the nucleolus of U87 and H1299 cells. Neither KPT-330 nor Act D increased 28S but tended to reduce 45/47S in U251 cells. Expressing a degradation-resistant form of XPO1 (C528S) partially reversed KPT-330-induced defects of rRNA processing (Fig. 5c–g). Consistently, KPT-330 suppressed nascent RNA synthesis in U87, U251, and H1299 cells (Fig. 5h). Not as reported recently^24^, KPT-330 did not significantly interfered rRNA nuclear export in any of the tested cells (Fig. 5i). These results suggest that KPT-330 inhibits rRNA processing, which will definitely influence ribosome complex.

### XPO1 reconstitution restores rRNA and Mcl-1 expression and drug resistance upon KPT-330 and A-1331852

**Fig. 6:**
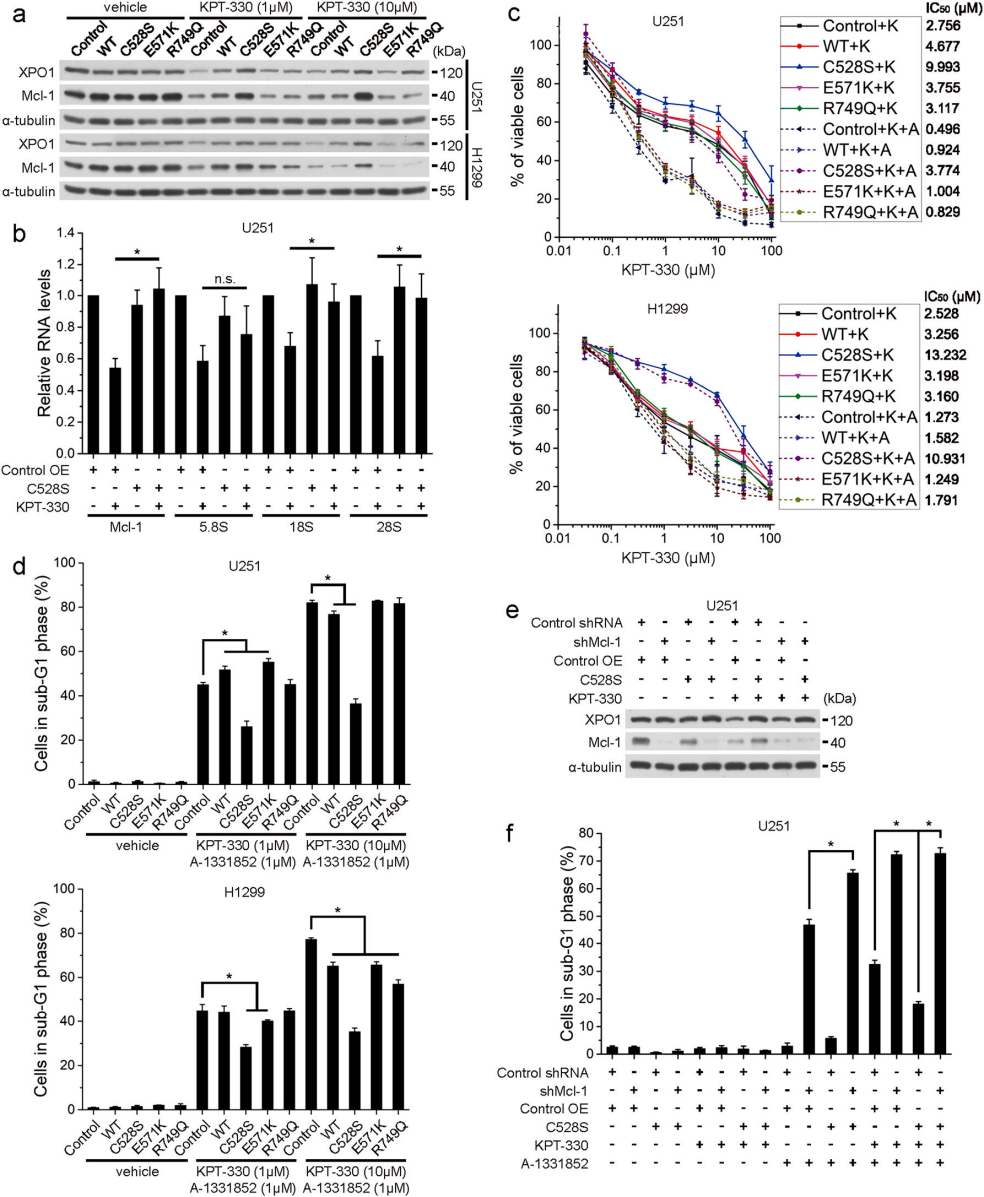
XPO1 reconstitution restores rRNA and Mcl-1 expression and drug resistance upon KPT-330 and A-1331852. **a** U251 and H1299 cells expressing different XPO1 variants were treated with 1 μM or 10 μM of KPT-330 for 24 h and subjected to western blot. **b** Real-time PCR analysis of indicated RNAs in C528S mutant-expressing U251 cells treated with KPT-330 (1 μM) for 24 h. (mean ± SEM, *n* = 5). *P < 0.05. **c** U251 and H1299 cells overexpressing different XPO1 variants were treated with indicated dose of KPT-330 (K) and with or without 1 μM of A-1331852 (A) for 48 h. Cell viability was measured by the MTT assay (mean ± SEM, *n* = 3 for U251 and *n* = 2 for H1299). IC_50_s were listed on the right. **d** U251 and H1299 cells expressing different XPO1 variants were treated with 1 μM or 10 μM of KPT-330 for 24 h and further with A-1331852 (1 μM) for 24 h, then subjected to flow cytometry analysis for the sub-G1 phase. (mean ± SD, *n* = 3). *P < 0.05. **e** Western blot of indicated proteins in shMcl-1- and/or C528S mutant-expressing U251 cells treated with KPT-330 (1 μM) for 24 h. **f** Cells treated as in **e** were further treated with A-1331852 (1 μM) for 24 h and subjected to flow cytometry for the sub-G1 phase (mean ± SD, *n* = 3). *P < 0.05. α-Tubulin was used as the loading control

To verify that XPO1 degradation upon KPT-330 contributes to Mcl-1 reduction and cancer cell apoptosis following KPT-330 and A-1331852 treatment, we reconstituted XPO1 expression by overexpressing wild-type XPO1, degradation-resistant mutant C528S, or recurrent hotspot mutant E571K or R749Q in U251 and H1299 cells. C528S mutant restored protein levels of XPO1 and Mcl-1 in cells treated with medium (1 μM) or high dose (10 μM) of KPT-330, while wild-type, E571K, and R749Q variants had weak effects (Fig. 6a). C528S mutant also restored rRNA and Mcl-1 mRNA in KPT-330-treated U251 cells (Fig. 6b). Accordingly, U251 and H1299 cells expressing C528S mutant but not cells expressing other XPO1 variants were resistant to KPT-330/A-13331852-induced viability reduction and apoptosis (Fig. 6c, d). However, C528S mutant failed to rescue KPT-330/A-13331852-induced apoptosis in Mcl-1-deficient U251 cells (Fig. 6e, f). Unexpectedly, C528S augmented apoptosis in A-1331852-treated Mcl-1-deficient cells (Fig. 6f). Bim was not involved in this effect since C528S did not enhanced Bim expression and Bim knockdown failed to abolish elevated apoptosis (Supplementary Fig. 5). Thus, these results demonstrate that KPT-330 targets XPO1 for degradation to disrupt rRNA and Mcl-1 expression, whereby it primes cancer cells to Bcl-xL inhibitor-induced apoptosis.

### Combination of KPT-330 and A-1331852 suppresses tumor growth in vivo

**Fig. 7:**
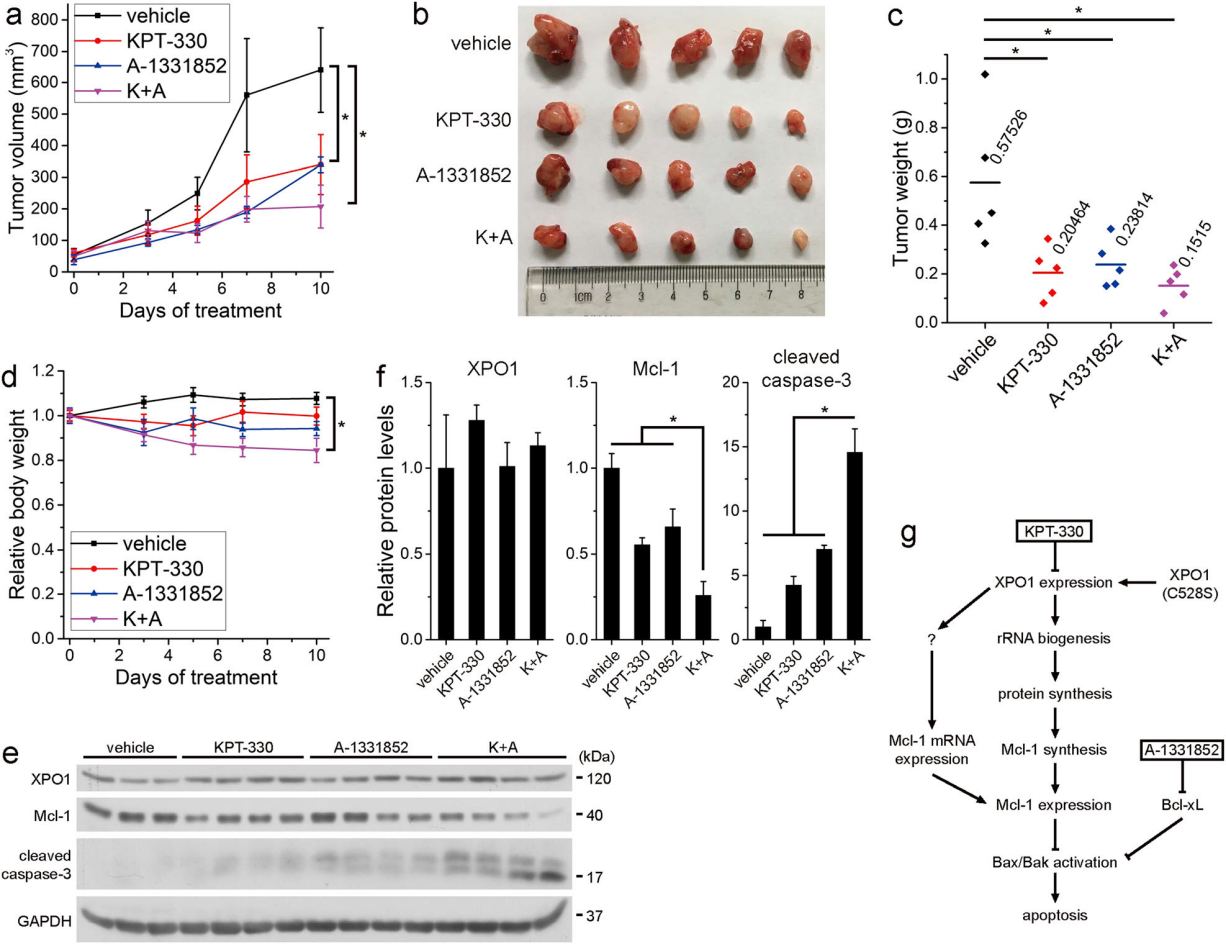
Combination of KPT-330 and A-1331852 suppresses tumor growth in vivo. **a** Tumor volume curves of H1299 xenografts in mice treated with vehicle, KPT-330, A-1331852, and both drugs. **b** Photograph showing tumor size in different groups 10 days after treatment. **c** Tumor weight of the corresponding H1299 xenografts. **d** Relative body weight curves of mice (mean ± SEM, *n* = 5). *P < 0.05. **e** Western blot analysis of xenografts isolated from mice treated as indicated. GAPDH was used as the loading control. **f** Grayscale ratio quantification of indicated proteins to GAPDH in **e** by Photoshop software (mean ± SEM, *n* = 3 for vehicle and *n* = 4 for other groups). *P < 0.05. **g** Schematic overview of the purposed pathway for KPT-330/A-1331852 combination therapy

Finally, to evaluate the anticancer activity of KPT-330/A-1331852 combination in vivo, we inoculated NOD-SCID mice with H1299 cells and treated them with KPT-330 (10 mg/kg, p.o., Monday, Wednesday, and Friday) and/or A-1331852 (25 mg/kg, p.o., every day) or vehicle for 10 days when tumor volume reached ~50 mm^3^. Exposure to KPT-330 or A-1331852 alone resulted in inhibition of tumor growth throughout the treatment and lower tumor weight in the end, while cotreatment with two drugs further suppressed tumor growth (Fig. 7a–c). However, the statistical difference of tumor volume or weight between cotreatment group and either monotherapy group was insignificant probably owing to good performance of either drug and relatively low synergistic effect of these drugs in H2199 cells in vitro (Fig. 7c). Mice in cotreatment group lost 15.5% of their body weight post treatment but were all alive (Fig. 7d). Despite not downregulating XPO1 level post treatment, KPT-330 generally maintained lower Mcl-1 level and, when combined with A-1331852, induced apoptosis in terms of caspase-3 cleavage (Fig. 7e, f). These results suggest that KPT-330/A-1331852 exerts anticancer effect in NSCLC xenografts and is tolerant in mice.

## Discussion

The clinical SINE compound KPT-330/selinexor is a specific and reversible XPO1 inhibitor, with oral bioavailability and tolerability. Preclinical studies have demonstrated its apoptotic-inducing effect in various types of cancers and different associated molecular mechanisms, like IκBα nuclear retention, NF-κB signaling inhibition, and survivin transcriptional inhibition^3,25^; nuclear accumulation of p53 and FOXO3a^26^. However, whether it directly regulates the mitochondrial apoptotic signaling leading to apoptosis remains elusive. SINE was shown to downregulate the antiapoptotic Bcl-2 protein Mcl-1 that counteracts MOMP, but no study scrutinized the underlying mechanism and associated phenotype^19–21^. Given that targeting Mcl-1, directly or indirectly, alone or combinatorial proved to be promising apoptosis-based anticancer therapeutic strategies^13,17,18,27,28^, we reckon that delineating the overlooked aspect is worthwhile and combination therapy based on such information may extend the application range and improve the performance of KPT-330 in cancer treatment. In this study, we show that KPT-330 suppresses nucleolar rRNA processing and total rRNA expression, which impedes global protein synthesis and Mcl-1 protein synthesis. It also decreases Mcl-1 mRNA expression by still unknown mechanism. Apoptosis in KPT-330-sensitive cancer cells depends on Mcl-1 reduction. Simultaneous inhibition of another apoptosis gatekeeper Bcl-xL using A-1331852 minimizes the sequestration of Bax and Bak and potently triggers MOMP and apoptosis. Synergistic effect of KPT-330/A-1331852 cotreatment is strong in cancer cells relatively more resistant to KPT-330 (U87, U118, U251, A549, and HeLa) and is moderate in KPT-330-sensitive cells (A172 and H1299) (Fig. 1e). Reconstituting XPO1 in cells by expressing the KPT-330-unbound C528S mutant restores the expression of rRNA and Mcl-1 and resistance to cotreatment of KPT-330 and A-1331852, while cells expressing recurrent hotspot mutant E571K and R749Q are as vulnerable to the cotreatment as those with wild-type XPO1 (Fig. 7g).

Life of Mcl-1 protein is short. Strategies like inhibition of mTORC1/4E-BP1 signaling to constrain cap-dependent global protein translation^13,17^ or signaling modifying Mcl-1 and coupling Mcl-1 to the ubiquitin-proteasome pathway^27^ commonly decrease Mcl-1 protein and suppress tumor growth. Coincide with the previous report that XPO1 inactivation dampened mTOR signaling^29^, we showed that KPT-330 inhibited mTORC1/4E-BP1 axis and Mnk1/eIF4E axis to diminish cap-dependent translation initiation activity, but dephosphorylation of 4E-BP1 and eIF4E lagged behind and was dispensable for Mcl-1 downregulation. Nor did we observe mTOR nuclear retention in our system (Fig. 3g) as before^29^. XPO1 regulates ribosome biogenesis. An iTRAQ analysis showed that SINE compound KPT-185 downregulated a series of ribosome proteins by ~10–27%^30^, while a recent study demonstrated that KPT-330 crippled nuclear export of 5S and 18S rRNA, ribosome assembly, and protein synthesis in glioblastoma cells^24^. However, our data challenge such explanation showing that KPT-330 basically did not alter the cytosolic/nuclear distribution of nucleolar processed 5.8S, 18S, and 28S rRNA, but rather reduced their total expression in glioblastoma cells, which were concordant with the decrease of RNA content and nascent RNA synthesis ability in these cells. One probable reason for the rRNA distribution difference is that we used U6 small nuclear RNA as housekeeping gene to normalize the relative nuclear RNA levels in real-time PCR analysis instead of Actin used in the previous study^24^. Consist with a previous study revealing that XPO1 inhibition using LMB disrupts rRNA synthesis and processing^31^, we found KPT-330 caused decreased pre-rRNAs while increased 28S levels in the nucleolus of U87 and H1299 cells, which further reduced total expression of mature rRNAs in glioblastoma cells. Despite enhanced 28S levels in the nucleolar, KPT-330 resulted in reduced nuclear 28S of glioblastoma cells, suggesting that KPT-330 suppressed nucleolar/nuclear export. Furthermore, KPT-330-mitigated RNA synthesis ability may reflect the fact but more likely reflects the difficulty of rRNA processing as 45S pre-rRNA is unstable if not processed and reduction of mature rRNAs impairs nascent RNA accumulation. Coincidence with more severe rRNA processing deficiency, RNA and protein synthesis rates, and RNA content were lower in H1299 cells than in U251 cells. We speculate that the defect of Actin expression may contribute to the failure of detecting rRNA downregulation in H1299 cells. The XPO1 ribosome export adaptor NMD3 facilitates XPO1 nucleolar localization and they cooperatively regulate rRNA synthesis and processing^31^. According to the TCGA database, *XPO1* and *NMD3* genes are frequently altered in lung squamous cell carcinoma and to a less extent in lung adenocarcinoma (Supplementary Fig. S3a). Accordingly, mRNAs of XPO1 and NMD3 are high in lung squamous cell carcinoma (Supplementary Fig. S3b). Lung cancer samples with *XPO1* alteration tend to express higher level of NMD3 (Supplementary Fig. S3c). In addition, NMD3 mRNA upregulation tends to accompany XPO1 mRNA upregulation (Supplementary Fig. S3d). Many coaltered genes in samples with XPO1 mRNA alteration function in RNA metabolism (Supplementary Fig. S3e). These bioinformatics information emphasize the key role of XPO1 in RNA metabolism and rRNA processing in cooperation with NMD3. Although mTORC1 controls ribosome biogenesis, including rRNA transcription and processing^32^, it is not the case here given that protein synthesis was attenuated 1 h after KPT-330 treatment^24^ and mTORC1 substrate 4E-BP1 was dephosphorylated 24 h after treatment (Fig. 4d). Besides, 45S pre-rRNA expression was hardly changed (Fig. 5b). Interaction of mTORC2 and ribosome improves the activity of mTORC2/Akt signaling and thereby activates mTORC1^33^. However, KPT-330 did not inhibit Akt phosphorylation (Supplementary Fig. S4). In addition, phosphorylation of ERK and p38, upstream regulators of Mnk1, were paradoxically upregulated (Supplementary Fig. S4). Therefore, expression inhibition of components like mTOR and Mnk1 rather than inactivation of upstream regulators possibly resulted in the suppression of mTORC1/4E-BP1 and Mnk1/eIF4E axes. Since these axes are less important in regulating Mcl-1 expression here, we did not explore the associated molecular mechanism.

We checked the status of several antiapoptotic, proapoptotic, and BH3 domain-only Bcl-2 proteins in H1299 cells following LMB or KPT-330 treatment. We observed a concurrence of band shift (probable phosphorylation) and downregulation of BimEL and downregulation of Mcl-1. Phosphorylation and degradation of Bim by kinases such as ERK upon anticancer treatment causes drug resistance^34^. We believe that Bim downregulation cooperates with or contributes to Bcl-xL/Bax interaction to make cancer cells adapt to XPO1 inhibition. Thus, fully neutralizing the activity of antiapoptotic Bcl-2 proteins can raise KPT-330 sensitivity. We chose Bcl-xL inhibitor A-1331852 to potentiate XPO1 efficacy considering the intolerance of Bcl-2/Bcl-xL inhibitor ABT-263 in the clinic and the primary role of Bcl-xL in apoptosis resistance in solid tumors after Mcl-1 inhibition^10–13^. Moreover, A-1331852 proves effective against solid tumors alone or in combination with other drugs in preclinical animal models and less toxic than Bcl-2 inhibitor or Bcl-2/Bcl-xL inhibitor to granulocytes like neutrophils^10,14^. Hence, it is reasonable and meaningful to evaluate the clinical application value of A-1331852 in terms of efficacy and safety.

In summary, we define the molecular basis of Mcl-1 reduction and apoptosis resistance upon KPT-330 treatment. Based on this mechanism, we develop a potential therapeutic strategy combining KPT-330 and A-1331852 against solid tumors. Such treatment is effective regardless the clinical relevant mutation status (E571K and R749Q) of XPO1. These findings provide a strong rationale for its further investigation in the clinic.

## Materials and methods

### Cell culture

Human glioblastoma cell lines A172, U87, U118, and U251 and cervical cancer cell line HeLa were cultured in DMEM supplemented with 10% fetal bovine serum (FBS), 1% nonessential amino acid, and 1% sodium pyruvate (Life technologies, Grand Island, USA). Human NSCLC cell lines H1299 and A549 were cultured in RPMI 1640 supplemented with 10% FBS. U87, U251, and H1299 were purchased in April 7, 2017 (purchase order, 85676), U118 and A172 were purchased in July 13, 2018 (purchase order, 115354), and HeLa was purchased in August 14, 2018 (purchase order, 117111) from cell bank of Chinese Academy of Sciences (Shanghai, China), where they were authenticated by means of STR profiling. STR profile report of A549 can be seen in the supplementary information. All cells were maintained under standard cell culture conditions at 37 °C and 5% CO_2_.

### Antibodies and reagents

Primary antibodies used in this study are listed as follows: antibodies against α-tubulin (HRP-conjugated) (HRP-66031) (ProteinTech Group, Wuhan, China), 4E-BP1 (9644), Bcl-xL (2764), Bak (12105) (for total Bak immunoprecipitation), Bax (5023) (for total Bax immunoprecipitation), Bim (2933), caspase-3 (9662), eIF4E (2067), eIF4G (2469), ERK (4695), GβL (3274), Mcl-1 (5453), MeCP2 (3456), Mnk1 (2195), mTOR (2983), Noxa (14766), p38 (8690), p-4E-BP1 (T37/46) (2855), p70S6K (2708), PARP (9532), p-Akt (S473) (4060), p-eIF4E (S209) (9741), p-ERK (T202/Y204) (4370), p-Mnk (T197/202) (2111), p-mTOR (S2448) (2972), p-p38 (T180/Y182) (4511), p-p70S6K (T389) (T389), Raptor (2280) (Cell Signaling Technology, Beverly, USA), Bax (6A7) (for active Bax immunoprecipitation) (556467) (BD Biosciences, San Jose, USA), Bak, NT (for active Bak immunoprecipitation) (06-536) (Merck Millipore, Darmstadt, Germany), LRPPRC (BS70542), XPO1 (BS70045) (Bioworld Technology, Nanjing, China), and GAPDH (KC-5G5) (Kangchen, Shanghai, China). Anti-mouse (7076) and anti-rabbit (7074) secondary antibodies (horseradish peroxidase-conjugated) were acquired from Cell Signaling Technology.

Reagents and kits used in this study are listed as follows: Selinexor (KPT-330), MG-132, z-VAD-FMK (Selleck, Shanghai, China), A-1155463, A-1331852, Actinomycin D (Act D), (Medchem Express, Monmouth Junction, USA), PMSF, propidium iodide, PVP (Sigma-Aldrich, St. Louis, USA), the CalPhos Mammalian Transfection Kit (TaKaRa Bio, Kusatsu, Japan), protease inhibitor cocktail, RNase A, Click-iT^TM^ RNA HCS Assays, Click-iT™ Plus OPP Protein Synthesis Assay Kit Alexa Fluor™ 488 picolyl azide, Lipofectamine 3000 Reagent, the PARIS™ Kit (Thermo Scientific, Waltham, USA), phosphatase inhibitor, thiazolyl blue tetrazolium bromide (Sangon, Shanghai, China), cell lysis buffer for western and IP, cycloheximide, the Mitochondrial Membrane Potential Assay Kit with JC-1, Nuclear and Cytoplasmic Protein Extraction Kit, RIPA (Beyotime, Nantong, China), LightCycler 480 SYBR Green I Master, protein A agarose, protein G agarose (Roche Diagnositics, Indianapolis, USA), Script Reverse Transcription Supermix (Bio-Rad, Berkeley, USA), immobilized 2′/3′-EDA-m^7^GTP (Jena Bioscience, Jena,Germany), the Dual-Luciferase^®^ Reporter Assay System (Promega Coporation, Madison, USA), Pluronic F-68 (Life technologies), and Phosal 50 PG (Lipoid, Ludwigshafen, German) were used in this study.

### Lentivirus-mediated gene transduction

Short hairpin RNAs (shRNAs) targeting human 4E-BP1, Bax, Bak and Mcl-1, and a scrambled (control) shRNA were inserted into the lentiviral vector pLKD-CMV-GFP-U6-shRNA. Coding DNA of human non-degradable Mcl-1 (T92A) mutant was inserted into pLOV-EF1a-eGFP, and coding DNAs of human eIF4E, XPO1 and XPO1 mutants C528S, E571K and R749Q were inserted into pCDH-CMV-MCS-EF1-copGFP. Lentiviral plasmids, gag/pol packaging vector, and VSVG encoding plasmid were transfected into 293 T cells using CalPhos Mammalian Transfection Kit according to the manufacturer’s protocol. Culture medium was harvested 48 h and 72 h after transfection and ultracentrifugated to obtain high-titer purified preparation. ShRNA sequences are listed in the Supplementary information.

### Western blot

After collection, cells were lysed in RIPA supplemented with PMSF, phosphatase inhibitor, and protease inhibitor cocktail. Western blot was carried out as previously described^35^. Grayscale of protein bands was analyzed by Photoshop CS4 software.

### MTT assay

Cell viability was measured by the MTT assay performed as previously described^35^.

### Flow cytometry assay

To measure apoptosis, fixed cells were stained with propidium iodide as previously described^35^. Mitochondrial membrane potential was determined using the Mitochondrial Membrane Potential Assay Kit with JC-1 according to the manufacturer’s protocol. After staining, cells were analyzed using a BD LSR II flow cytometer. Sub-diploid cells were considered apoptotic. The proportion of sub-dipliod cells were analyzed by FlowJo 7.6.1 software.

### Immunoprecipitation

Cells were lysed on ice in cell lysis buffer for western and IP (20 mM Tris (pH 7.5), 150 mM NaCl, 1% Triton X-100). Lysates were adjusted to have equal protein concentrations and incubated with indicated antibodies overnight, and with additional protein A agarose or protein G agarose at 4 °C for 3 h. Precipitates were washed three times with lysis buffer before adding SDS-PAGE loading buffer and denaturation. Precipitates and lysates were then subjected to western blot.

### Nucleoli isolation

Nucleoli were prepared as previously described^36^. Briefly, Cells detached with trypsin/EDTA were lysed in hypotonic buffer (10 mM Hepes, PH 7.5, 10 mM KCl, 1.5 mM MgCl_2_, 0.5 mM DTT) with a Dounce tissue homogenizer until at least 90% of the nuclei had been released. The nuclear fractions were separated from cytoplasmic fractions by centrifugation at 300 *g* for 5 min at 4 °C and purified by sucrose cushion (0.35 M sucrose, 0.5 mM MgCl_2_) centrifugation. The nucleoli were prepared by sonicating the resuspended purified nuclear fractions in 0.35 M sucrose and 0.5 mM MgCl_2_ solution and purifying with sucrose cushion (0.88 M sucrose, 0.5 mM MgCl_2_) centrifugation.

### RNA extraction, real-time PCR, and RNA fragment analysis

RNA was extracted, reverse transcribed, and analyzed by quantitative real-time PCR as previously described^35^. Primer sets for PCR are listed in the supplementary information. RNA fragment analyses were performed with the Fragment Analyzer™ Automated CE System (Agilent formerly Advanced Analytical Technologies, California, USA) following the manufacturer’s protocol.

### Nascent RNA and protein synthesis assay

Cells were plated in 96-well culture plates. After drug treatment, cells were incubated with 1 μM 5-ethynyl uridine (EU) for 1 h to probe nascent RNA or with 20 μM O-propargyl-puromycin (OPP) for 0.5 h to probe nascent protein. EU and OPP detection were performed according to the manufacturer’s protocols. Fluorescence was measured by multi-mode microplate reader. FITC intensity was normalized against DNA-bound NuclearMask Blue stain intensity as the internal control.

### Bicistronic luciferase reporter assay

The luciferase reporter assay was conducted using a dual-luciferase reporter assay system according to the manufacturer’s protocol. Cells were transfected with a bicistronic luciferase reporter plasmid, pcDNA3-rLuc-polioIRES-fLuc, directing cap-dependent translation of *Renilla* luciferase gene and cap-independent polioIRES-dependent translation of the firefly luciferase gene^37^ using Lipofectamine 3000. After 24 h transfection, Cells were treated with 1 μM KPT-330 for 24 h. Luminescence was measured by multi-mode microplate reader. The renilla/firefly luciferase luminescence was calculated for cap-dependent translational activity.

### Cap-binding assay

Cell lysates were incubated with m^7^GTP agarose at 4 °C for 3 h to capture eIF4E and its binding proteins. Following procedures were performed in the same procedure as immunoprecipitation.

### Isolation of cytosolic and nuclear RNA

Cytosolic and nuclear RNAs were separated using the PARIS kit according to the manufacturer’s protocol.

### Human NSCLC xenografts study

Male NOD-SCID mice (5 weeks, Shanghai Lingchang Bioscience Company, China) were maintained in the pathogen-free environment. All experimental procedures were approved by the Institutional Animal Care and Use Committee of the Institute of Neuroscience, Chinese Academy of Science. H1299 cells (9 × 10^6^) in 150 μl serum-free DMEM were injected under the skin of mice. When average tumor volume reached ~50 mm^3^, mice were randomized into four groups (*n* = 5 per arm) and treated with KPT-330 (10 mg/kg, Monday, Wednesday, and Friday of every week) and/or A-1331852 (25 mg/kg, daily) by oral gavage. The vehicle for KPT-330 was 18.5% DMSO, 0.6% Pluronic F-68, and 0.6% PVP in water. The vehicle for A-1331852 was 2.5% DMSO, 10% ethanol, 27.5% PEG 300, and 60% Phosal 50 PG^14^. Tumor volume was calculated using the formula: *V* = 0.5 × length × width^2^. The treatment lasted for 10 days. One day after the final drug administration, mice were euthanized and tumors were isolated.

### Statistical analysis

OriginPro 8 software (OriginLab Corporation, Northampton, USA) was used for data analysis and graphing. Results are expressed as mean ± SD or mean ± SEM. The one-way ANOVA test was used for nucleolar rRNAs processing tests and the two-tailed unpaired *t*-test was used for other tests to determine significant differences between the mean values of groups, with statistical significance defined as *p* < 0.05.

## Supplementary information

Supplementary Information
