# Supplementary material for: XPO1 inhibitor KPT-330 synergizes with Bcl-xL inhibitor to induce cancer cell apoptosis by perturbing rRNA processing and Mcl-1 protein synthesis
Source: Cell Death Dis. 2019 May 21;10(6):395. doi: 10.1038/s41419-019-1627-9 (PMC6529444; doi:10.1038/s41419-019-1627-9)
Supplement: Supplementary file 1 — Supplementary Information [file 41419_2019_1627_MOESM1_ESM.pdf]

Supplementary Information

The sequence of shRNAs used were as follows: shBax, CCGGGCTCTGAGCAGATCATGAATTCAAGAGATTCATGATCTGCTCAGAGCTTTTTTG; shBak, CCGGCCCATTCACTACAGGTGAATTCAAGAGATTCACCTGTAGTGAATGGGTTTTTTG; shBim, CCGGGACCGAGAAGGTAGACAATTGCTCGAGCAATTGTCTACCTTCTCGGTCTTTTTTG; shNoxa, CCGGGCAAGAACGCTCAACCGAGTTCAAGAGACTCGGTTGAGCGTTCTTGCTTTTTTG; sh4E-BP1, CCGGGCCAGGCCTTATGAAAGTGATCTCGAGATCACTTTCATAAGGCCTGGCTTTTTT; shMcl-1, CCGGCCCTAGCAACCTAGCCAGAAACTCGAGTTTCTGGCTAGGTTGCTAGGGTTTTTTG; scrambled shRNA, CCGGTTCTCCGAACGTGTCACGTTTCAAGAGAACGTGACACGTTCGGAGAATTTTTTG.

Primer sets used for real-time PCR were as follows: MCL1, 5’-TGCTTCGGAAACTGGACATCA-3’, 5’-TAGCCACAAAGGCACCAAAAG-3’; ACTB, 5’-CATGTACGTTGCTATCCAGGC-3’, 5’-CATGTACGTTGCTATCCAGGC-3’; RNA5S, 5’-GATCTCGGAAGCTAAGCAGG-3’, 5’-AAGCCTACAGCACCCGGTAT-3’; RNA5-8S, 5’-CTCTTAGCGGTGGATCACTC-3’, 5’-GACGCTCAGACAGGCGTAG-3’; RNA18S, 5’-CAGCCACCCGAGATTGAGCA-3’, 5’-TAGTAGCGACGGGCGGTGTG-3’; RNA28S, 5’-TCATCAGACCCCAGAAAAGG-3’, 5’-GATTCGGCAGGTGAGTTGTT-3’; RNA45S, 5’-TGTCAGGCGTTCTCGTCTC-3’, 5’-AGCACGACGTCACCACATC-3’; U6, 5’-CTCGCTTCGGCAGCACA-3’, 5’-AACGCTTCACGAATTTGCGT-3’.

Supplementary Figures

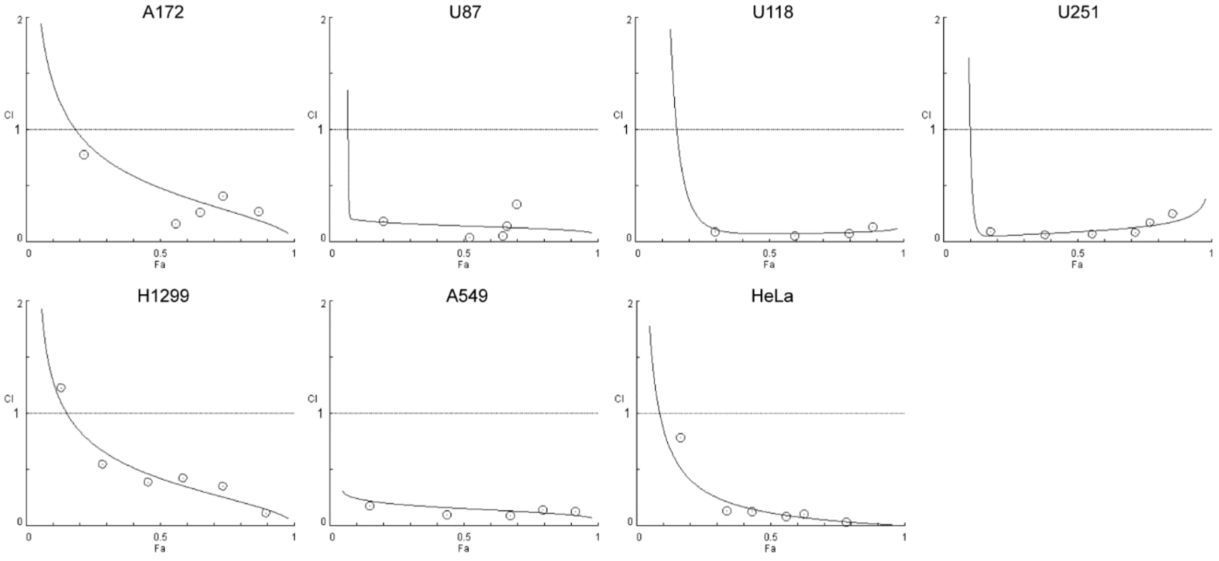

Supplementary Fig. S1 CI versus factional effect (Fa) plot showing the combination effect of KPT-330 with A-1331852 in seven cell lines are illustrated in the isobolograms. CI<0.9 is considered as synergistic, 0.9<CI<1.1 as additive and CI>1.1 as antagonistic.

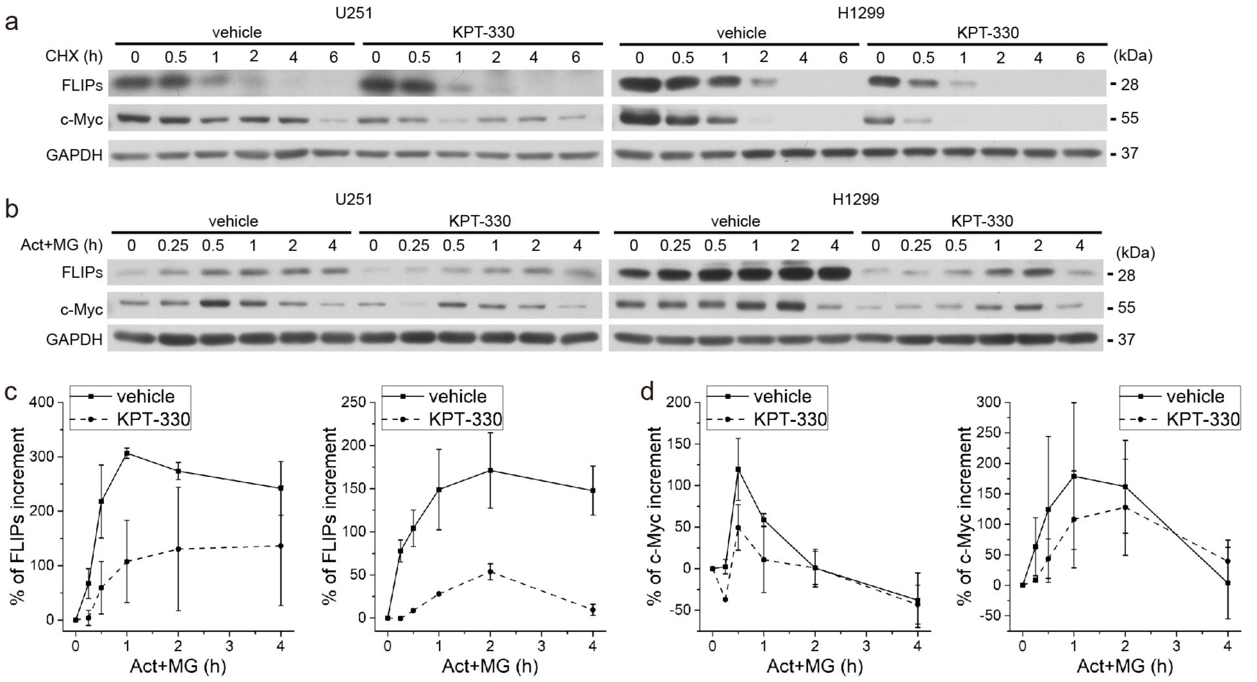

Supplementary Fig. S2 KPT-330 suppresses FLIPs and c-Myc protein synthesis. (**a**) CHX (100 μg/ml) pulse-chase assay in U251 and H1299 cells treated KPT-330 (1 μM) for 24 h. (**b**) Western blot analysis of FLIPs and c-Myc protein synthesis in U251 and H1299 cells treated with KPT-330 (1 μM) for 24 h, and further with Act D (Act) (5 μg/ml) and MG-132 (MG) (25 μM) for indicated time periods. Quantification of grayscale ratio of FLIPs/GAPDH and c-Myc/GAPDH by Photoshop software were shown in **c** and **d** (mean±SEM, n=2) respectively.

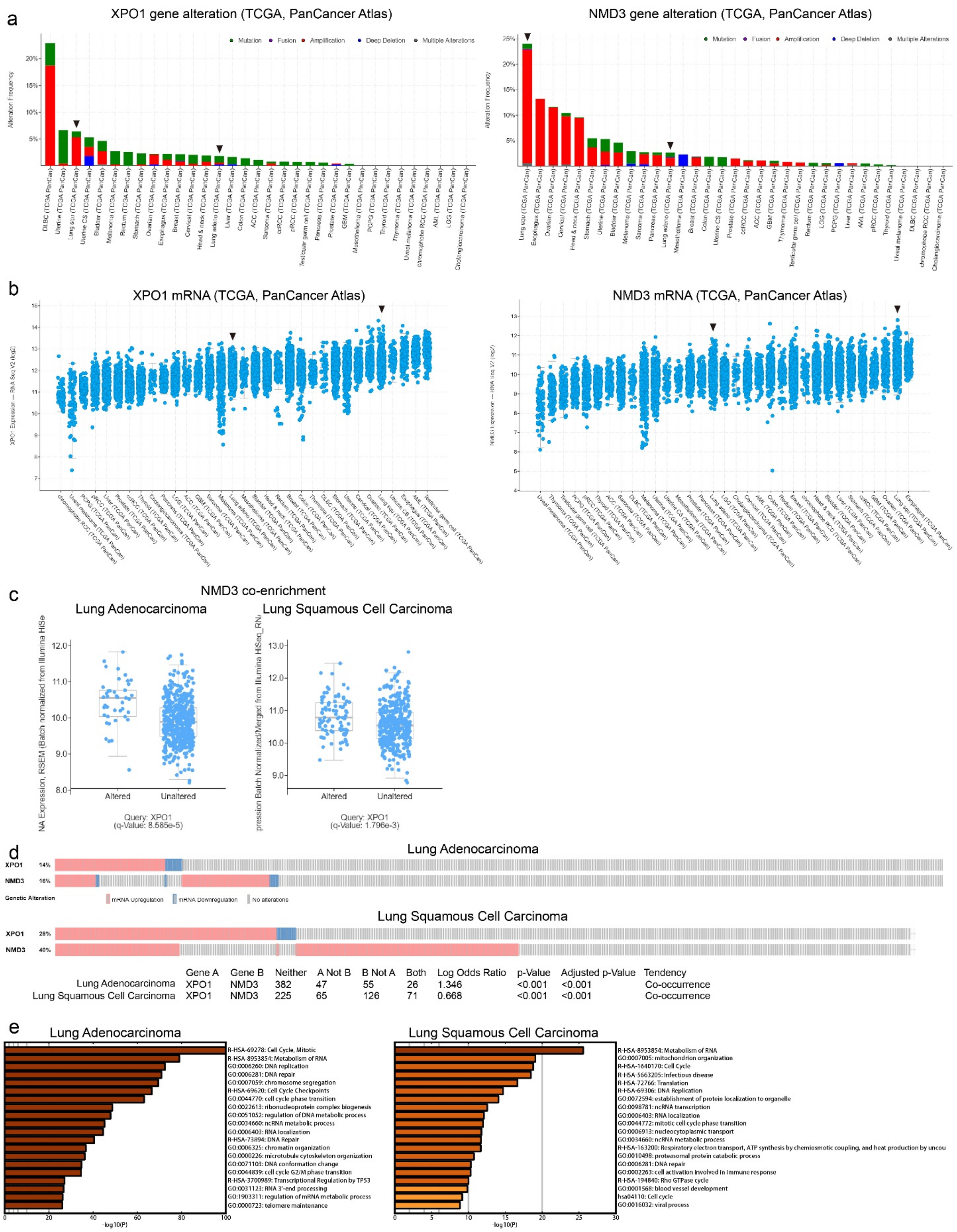

Supplementary Fig. S3 (**a**) Bioinformatics analysis of gene alteration of *XPO1* and its nuclear export adaptor *NMD3* in various types of cancers based on the PanCancer Atlas program in the TCGA database. Amplification of both *XPO1* and *NMD3* are common in lung squamous cell carcinoma. (**b**) Relative mRNA levels of XPO1 and NMD3 in various types of cancers. (**c**) NMD3 mRNA levels in lung adenocarcinoma and lung squamous cell carcinoma with or without *XPO1* gene alteration. (**d**) Concurrent mRNA alteration of XPO1 and NMD3 in these two types of cancers. mRNA expression z-score threshold ± 1.5. (**e**) Pathways analysis showing many co-altered genes in these lung cancer samples with XPO1

mRNA alteration function in RNA metabolism. XPO1 mRNA expression z-score threshold ± 2.0. Genetic and mRNA alterations of XPO1 and NMD3 were analyzed using the cBioPortal database (www.cbioportal.org). Pathway analysis was performed using Metascape (metascape.org).

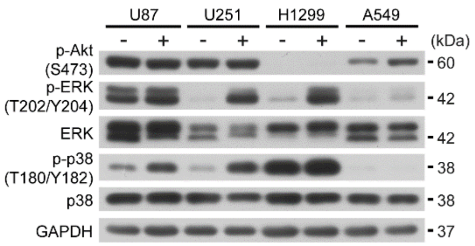

Supplementary Fig. S4 U87, U251, H1299 and A549 cells were treated with KPT-330 (1 μM) for 48h and subjected to western blot. GAPDH was used as the loading control.

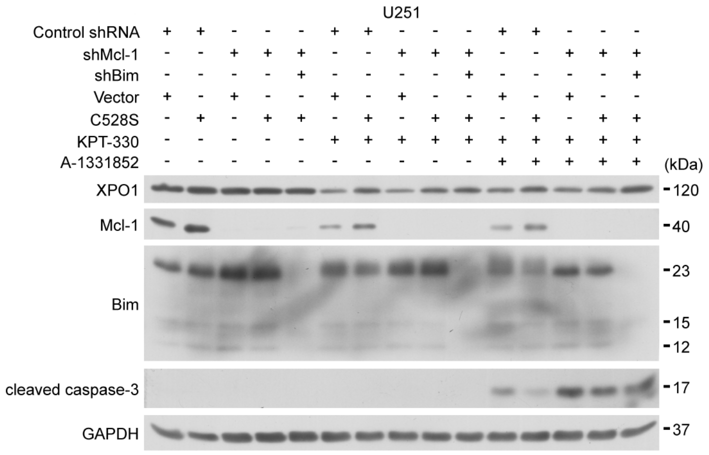

Supplementary Fig. S5 U251 cells expressing shMcl-1, shBim, XPO1(C528S) were treated with KPT-330 (1 μM) for 24h and further with A-1331852 (1 μM) for 6 h, then subjected to western blot. GAPDH was used as the loading control.
